# A conserved role of EIN3 in the development of rooting structures in land plants

**DOI:** 10.1093/plphys/kiag104

**Published:** 2026-05-13

**Authors:** Dongdong Kong, Jie Meng, Yidong Wang, Jiao Liu, Wenxiu Cui, Legong Li, Chuanli Ju

**Affiliations:** College of Life Sciences, Capital Normal University, and Beijing Key Laboratory of Plant Gene Resources and Biotechnology for Carbon Reduction and Environmental Improvement, Beijing 100048, China; College of Life Sciences, Capital Normal University, and Beijing Key Laboratory of Plant Gene Resources and Biotechnology for Carbon Reduction and Environmental Improvement, Beijing 100048, China; College of Life Sciences, Capital Normal University, and Beijing Key Laboratory of Plant Gene Resources and Biotechnology for Carbon Reduction and Environmental Improvement, Beijing 100048, China; College of Life Sciences, Capital Normal University, and Beijing Key Laboratory of Plant Gene Resources and Biotechnology for Carbon Reduction and Environmental Improvement, Beijing 100048, China; College of Life Sciences, Capital Normal University, and Beijing Key Laboratory of Plant Gene Resources and Biotechnology for Carbon Reduction and Environmental Improvement, Beijing 100048, China; College of Life Sciences, Capital Normal University, and Beijing Key Laboratory of Plant Gene Resources and Biotechnology for Carbon Reduction and Environmental Improvement, Beijing 100048, China; College of Life Sciences, Capital Normal University, and Beijing Key Laboratory of Plant Gene Resources and Biotechnology for Carbon Reduction and Environmental Improvement, Beijing 100048, China

## Abstract

The ethylene signaling transcription factor EIN3 regulates rooting structure development in a conserved manner in land plants.

Dear Editor,

Land plants are anchored to their growth substrate through specialized rooting structures. In the moss *Physcomitrium patens* (*P. patens*; Rensing et al. 2020), a representative of one of the most basal land plant lineages, this function is carried out by rhizoids (Szövényi et al. 2019). Rhizoids, emerging from the base and mid-stem of the leafy gametophore, are multicellular, tip-growing filaments that resemble root hairs in vascular plants in both form and function (Szövényi et al. 2019). Root hairs, in contrast, are tubular protrusions of single epidermal cells located in the maturation zone of angiosperm roots. Both rhizoids and root hairs are involved in substrate anchorage and nutrient uptake (Honkanen and Dolan 2016). Previous studies have revealed conserved transcriptional regulators, including basic helix–loop–helix (bHLH) transcription factors, that govern root hair formation in *Arabidopsis thaliana* (Arabidopsis) and rhizoid development in *P. patens* (Menand et al. 2007; Tam et al. 2015). However, the extent to which phytohormones contribute to the regulation of these rooting structures remains insufficiently explored.

Ethylene, a gaseous phytohormone, regulates diverse aspects of plant growth and stress responses (Yang and Hoffman 1984). In Arabidopsis, ethylene promotes root hair development through the coordinated action of the master regulator ETHYLENE-INSENSITIVE 3 (EIN3)/EIN3-LIKE 1 (EIL1) and the root hair promoting transcription factors ROOT HAIR DEFECTIVE 6 (RHD6)/RHD6-LIKE 1 (RSL1) (Feng et al. 2017). Ethylene signaling is thought to have originated before the emergence of terrestrial plants over 450 million years ago (Ju et al. 2015). In *P. patens*, ethylene regulates 3D growth (Wang et al. 2024)—a key innovation that facilitated terrestrial adaptation (Moody 2019). Rhizoids, as specialized filamentous outgrowths at the plant–soil interface, also represent a major innovation of early land plants (Szövényi et al. 2019). Yet, whether ethylene regulates rhizoid development has remained unclear. In this study, we investigated the potentially conserved role of *P. patens* EIN3 homologs in regulating rhizoid development in *P. patens* and root hair growth in Arabidopsis.

To identify *P. patens* homologs of Arabidopsis EIN3 (AtEIN3, AT3G20770), we performed BLAST searches against the *P. patens* genome (v3.3) using the Phytozome database (https://phytozome-next.jgi.doe.gov/). Two homologs were identified, designated *PpEIN3a* (Pp3c7_9970) and *PpEIN3b* (Pp3c11_15260). PpEIN3a and PpEIN3b proteins share 38.2% and 37.6% identity with AtEIN3, respectively, and they are highly conserved with AtEIN3 in regions predicted to be the nuclear localization sequence and the core DNA-binding domain (Figure S1; Song et al. 2015). PpEIN3a and PpEIN3b exhibit 81.4% identity and 92.5% similarity to each other.

To investigate the roles of 2 *PpEIN3* genes in rhizoid development, we carried out expression pattern analysis and generated the knockout mutants and overexpression (OE) lines in *P. patens* for phenotypic characterization (see Supplementary materials and methods and Table S1). Both *PpEIN3a* and *PpEIN3b* genes were expressed in protonemata as well as the rhizoid, caulidium, and phyllid of the gametophore (Fig. 1a). Given the high similarity between the 2 PpEIN3 proteins, we created *Ppein3a/b* double mutants disrupting the function of both genes (Figure S2) via CRISPR-Cas9 gene editing (Lopez-Obando et al. 2016). *Ppein3a/b* mutants exhibited an approximately 50% reduction in rhizoid length compared with the wild type (WT) (Fig. 1b and c), based on the measurement of the 5 longest rhizoids per gametophore, indicating the involvement of 2 *PpEIN3* genes in rhizoid development. The shoot height of the mutants' gametophores was also reduced compared with the WT (Figure S3). We further examined whether each gene alone could influence rhizoid development by generating stable OE lines of *PpEIN3a* and *PpEIN3b* in *P. patens*. Homologous recombination confirmed the integration of each construct, and PCR-based genotyping validated the transgenic lines (Figure S4). Transcript abundance analysis confirmed strong expression of the transgenes (Fig. 1d and e). Both *PpEIN3*a OE and *PpEIN3b* OE lines exhibited enhanced rhizoid development compared with WT, characterized by the obviously longer rhizoids (Fig. 1f to i). The 2 *PpEIN3a* OE lines displayed a stronger phenotype than the 2 *PpEIN3b* OE lines (Fig. 1f to i), presumably due to the higher levels of *PpEIN3a* expression or the more dominant role of *PpEIN3a* in rhizoid development. Additionally, both sets of transgenic lines showed shorter shoot height than the WT (Figure S5). These findings demonstrate that both EIN3 homologs positively regulate rhizoid growth in *P. patens*, in addition to their previously reported involvement in submergence response (Karim et al. 2025).

**Figure 1:**
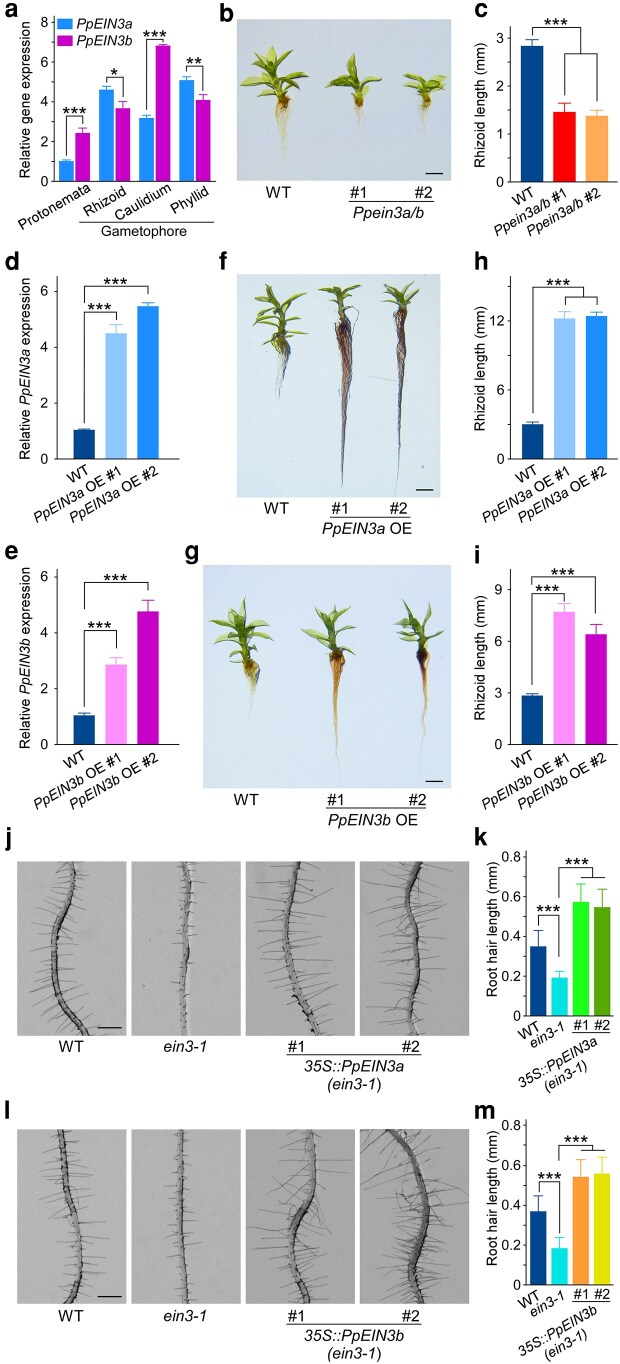
Functional conservation of *PpEIN3a* and *PpEIN3b* with *AtEIN3* in regulating plant rooting structures. **a)** Relative expression of *PpEIN3a* and *PpEIN3b* in the protonemata and gametophore of *P. patens*. Protonemal tissue and gametophores were respectively collected at 7 days and 35 days after inoculation of the protonemata on BCD medium. Data represent mean ± SD (*n* = 3). *, **, and *** indicate *P* ≤ 0.05, *P* ≤ 0.01, and *P* ≤ 0.001, respectively, between the 2 genes in the same tissue (Student's *t*-test). **b)** Rhizoid phenotypes of WT and *Ppein3a/b* double mutants. Gametophore apices of uniform size were cultured on BCD medium for 35 days; representative image is shown. Scale bar = 1 mm. **c)** Quantification of rhizoid length in genotypes shown in **b)**. For each genotype, the top 5 longest rhizoids per gametophore were measured, with a total of 75 rhizoids measured (*n* = 15 gametophores). Data represent mean ± SD (*n* = 75). *** indicates statistical significance relative to WT at *P* ≤ 0.001 (Student's *t*-test). **d** and **e)** Expression analyses of *PpEIN3a* in *PpEIN3a* OE lines and *PpEIN3b* in *PpEIN3b* OE lines of *P. patens*. Gametophores at 35 days after inoculation on BCD medium were used for the analysis. Data represent mean ± SD (*n* = 3). *** indicates *P* ≤ 0.001 compared with WT (Student's *t*-test). **f** and **g)** Rhizoid phenotypes of *PpEIN3a* OE and *PpEIN3b* OE transgenic plants. Gametophore apices were cultured on BCD medium for 35 days; representative images are shown. Scale bars = 1 mm. **h** and **i)** Quantification of rhizoid length in genotypes shown in **f)** and **g)**, respectively. Data represent mean ± SD (*n* = 75 rhizoids from 15 gametophores). *** indicates statistical significance relative to WT at *P* ≤ 0.001 (Student's *t*-test). **j)** Root hair phenotypes of *Arabidopsis* WT, *ein3-1* mutant, and *35S::PpEIN3a* (in *ein3-1* background) lines. Seedlings were grown on half-strength MS medium for 5 days; representative images are shown. Scale bar = 0.5 mm for all images in **j)**. **k)** Quantification of root hair length in genotypes shown in **j)**. Data are mean ± SD (*n* ≥ 15 seedlings). *** indicates *P* ≤ 0.001 compared with WT or *ein3-1* mutant (Student's *t*-test). **l)** Representative root hair images of *35S::PpEIN3b* (in *ein3-1* background) lines. Scale bar = 0.5 mm for all images in **l)**. **(m)** Quantification of root hair length in genotypes shown in **l)**. Data are mean ± SD (n ≥ 15 seedlings). *** indicates *P* ≤ 0.001 compared with WT or *ein3-1* mutant (Student's *t*-test).

To assess functional conservation across plant groups, we introduced *PpEIN3a* and *PpEIN3b* into the Arabidopsis *ein3-1* loss-of-function mutant, which is defective in root hair development (Feng et al. 2017). Expression of *PpEIN3a* or *PpEIN3b* under the CaMV 35S promoter was confirmed in independent transgenic lines (Figure S6). Both transgenes rescued the *ein3-1* phenotype, restoring root hair length beyond WT levels (Fig. 1j to m). These results indicate that *PpEIN3a* and *PpEIN3b* can functionally substitute for *AtEIN3* in Arabidopsis, supporting a conserved role of *EIN3* genes in controlling the development of rhizoids and root hairs in land plants.

In Arabidopsis, AtEIN3 physically interacts with AtRHD6 (Feng et al. 2017), forming the molecular basis of ethylene-mediated root hair development. To test whether a similar mechanism operates in moss, we cloned 2 *P. patens RHD6-LIKE* (*PpRSL*) genes, *PpRSL1* and *PpRSL2*, which were previously reported to be functionally equivalent to *AtRHD6* (Menand et al. 2007). Yeast 2-hybrid assays showed that PpEIN3a interacts with both PpRSL1 and PpRSL2 (Fig. 2a), and PpEIN3b also interacts with both PpRSL proteins (Fig. 2b). Bimolecular fluorescence complementation assays further confirmed these interactions *in planta* (Fig. 2c and d). These findings indicate that the EIN3–RSL interaction is conserved between *P. patens* and Arabidopsis.

**Figure 2:**
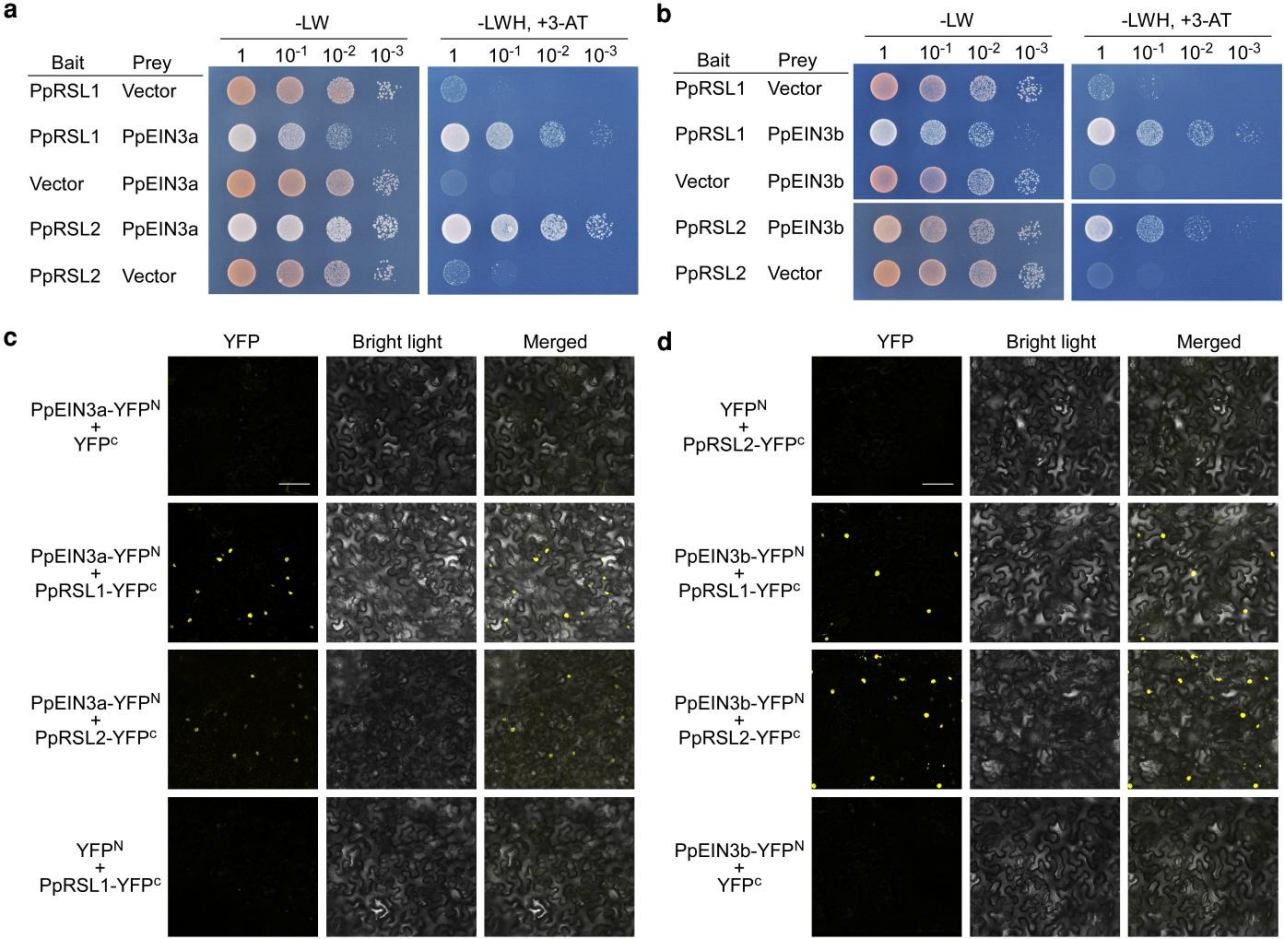
PpEIN3a and PpEIN3b physically interact with PpRSL1 and PpRSL2. a) Yeast 2-hybrid assays showing interaction of PpEIN3a with PpRSL1 and PpRSL2. Growth on -LW medium indicates viability; growth on -LWH medium supplemented with 1.5 mM 3-AT indicates protein–protein interaction. b) Interaction of PpEIN3b with PpRSL1 and PpRSL2 in yeast 2-hybrid assays. c) Bimolecular fluorescence complementation assays showing interactions of PpEIN3a with PpRSL1 and PpRSL2 in *N. benthamiana* leaf epidermal cells. Constructs were transiently co-expressed and visualized using confocal microscopy. Scale bar = 100 µm for all images in c). d) Bimolecular fluorescence complementation assays showing interactions of PpEIN3b with PpRSL1 and PpRSL2. Scale bar = 100 µm for all images in d).

In conclusion, our study provides genetic and molecular evidence that EIN3 homologs in bryophytes share a similar function with their angiosperm counterparts to regulate the development of rooting structures. Together with prior findings that PpRSL1 and PpRSL2 regulate rhizoid growth (Menand et al. 2007), our results support the conservation of the EIN3/EIL1–RHD6/RSL regulatory module in land plants. Our findings suggest that the functional role of EIN3 in controlling rooting structures was established in the common ancestor of mosses and seed plants.

## Supplementary Material

kiag104_Supplementary_Data

## Data Availability

All data can be found in the manuscript and in the supporting information.
