# Supplementary material for: A conserved role of EIN3 in the development of rooting structures in land plants
Source: Plant Physiol. 2026 May 13;201(1):kiag104. doi: 10.1093/plphys/kiag104 (PMC13170697; doi:10.1093/plphys/kiag104)
Supplement: kiag104_Supplementary_Data [file kiag104_supplementary_data.pdf]

Supplementary figures

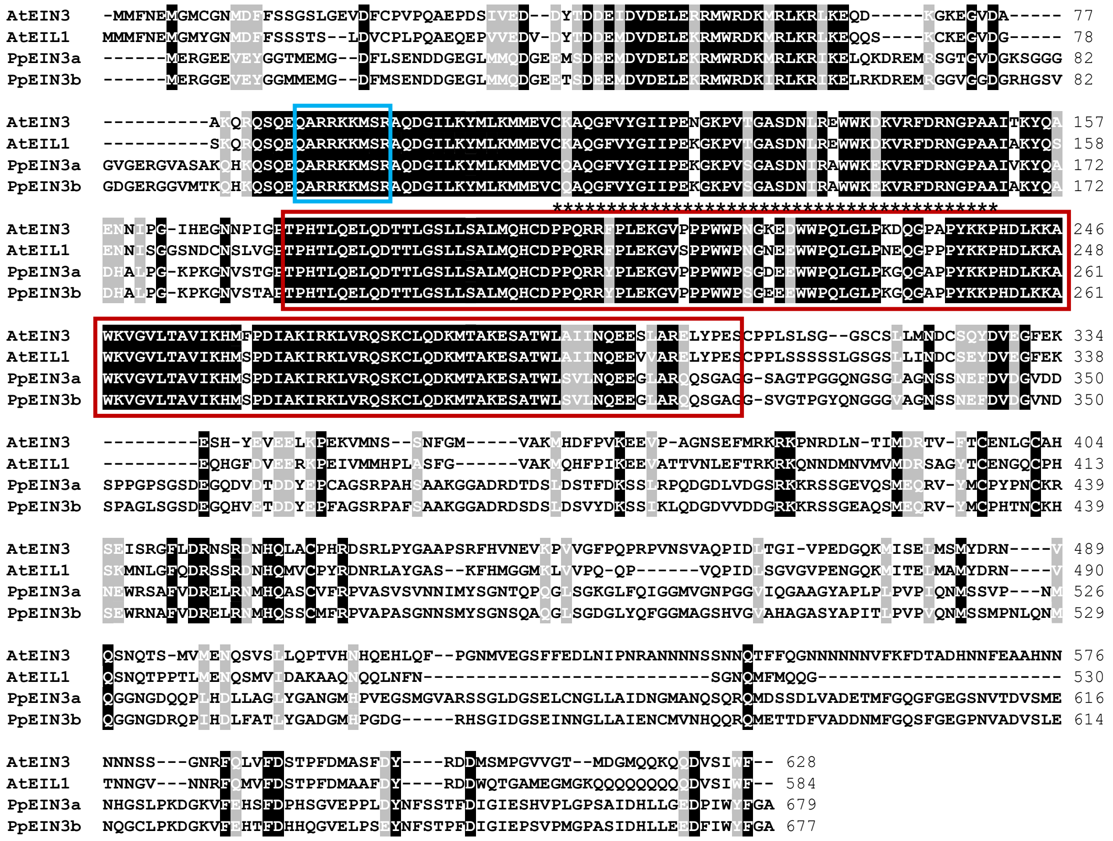

Supplementary Fig. S1. Sequence alignment of *Arabidopsis* EIN3 (AtEIN3) and EIL1 (AtEIL1) with PpEIN3a and PpEIN3b. Identical and similar amino acids are shaded in black and grey, respectively. Blue and red boxes indicate the regions corresponding to the predicted nuclear localization sequence and the core DNA-binding domain of AtEIN3 (Song et al., 2015). Residues marked by * show the proline-rich region.

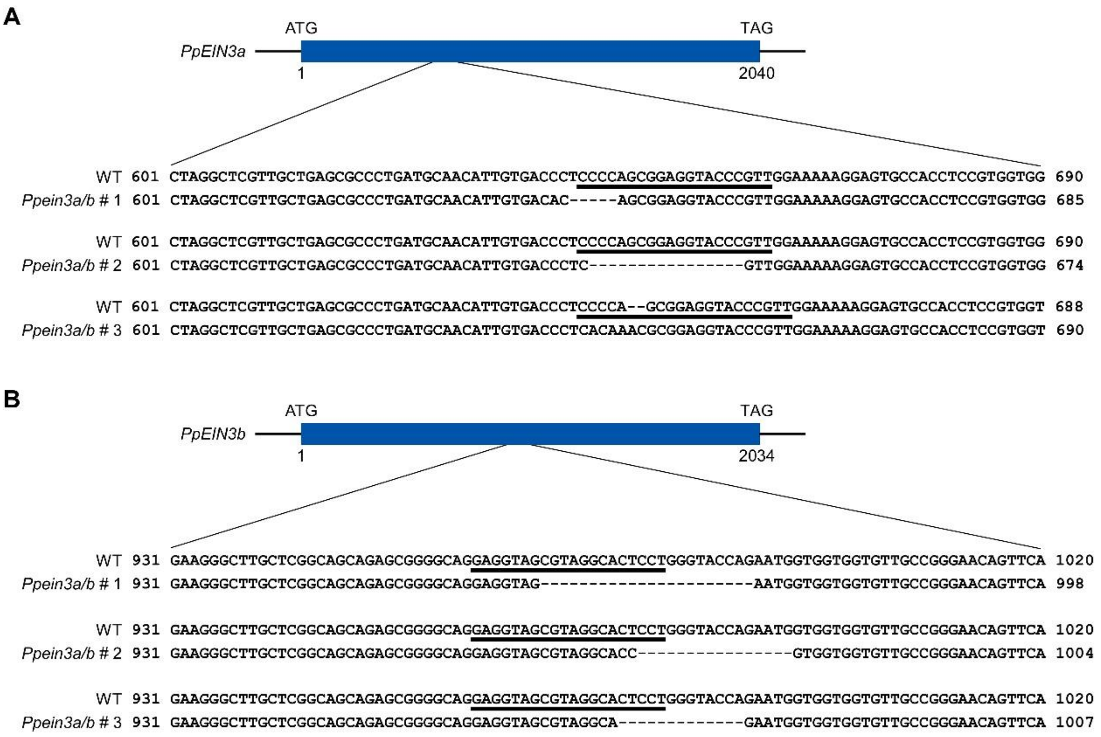

Supplementary Fig. S2. Mutations in the *Ppein3a/b* mutants. (A) Genomic structure of *PpEIN3a* and partial sequencing result showing mutations of *PpEIN3a* in three independent lines of the *Ppein3a/b* mutants. The blue box indicates the exon. The line underneath WT sequence indicates the position of sgRNA sequence for *PpEIN3a* gene. **(B)** Genomic structure of *PpEIN3b* and partial sequencing result indicating mutations of *PpEIN3b* in three independent lines of the *Ppein3a/b* mutants. The blue box indicates the exon. The line underneath WT sequence indicates the position of sgRNA sequence for *PpEIN3b* gene.

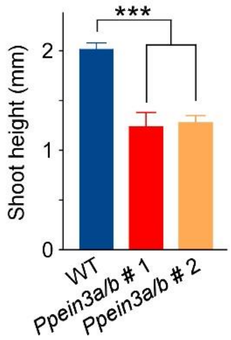

Supplementary Fig. S3. Quantification of gametophore shoot height in WT and *Ppein3a/b* double mutants. Data represent mean ± SD (n = 15 gametophores). *** indicates statistical significance relative to WT at $P \leqslant 0.001$ (Student's t-test).

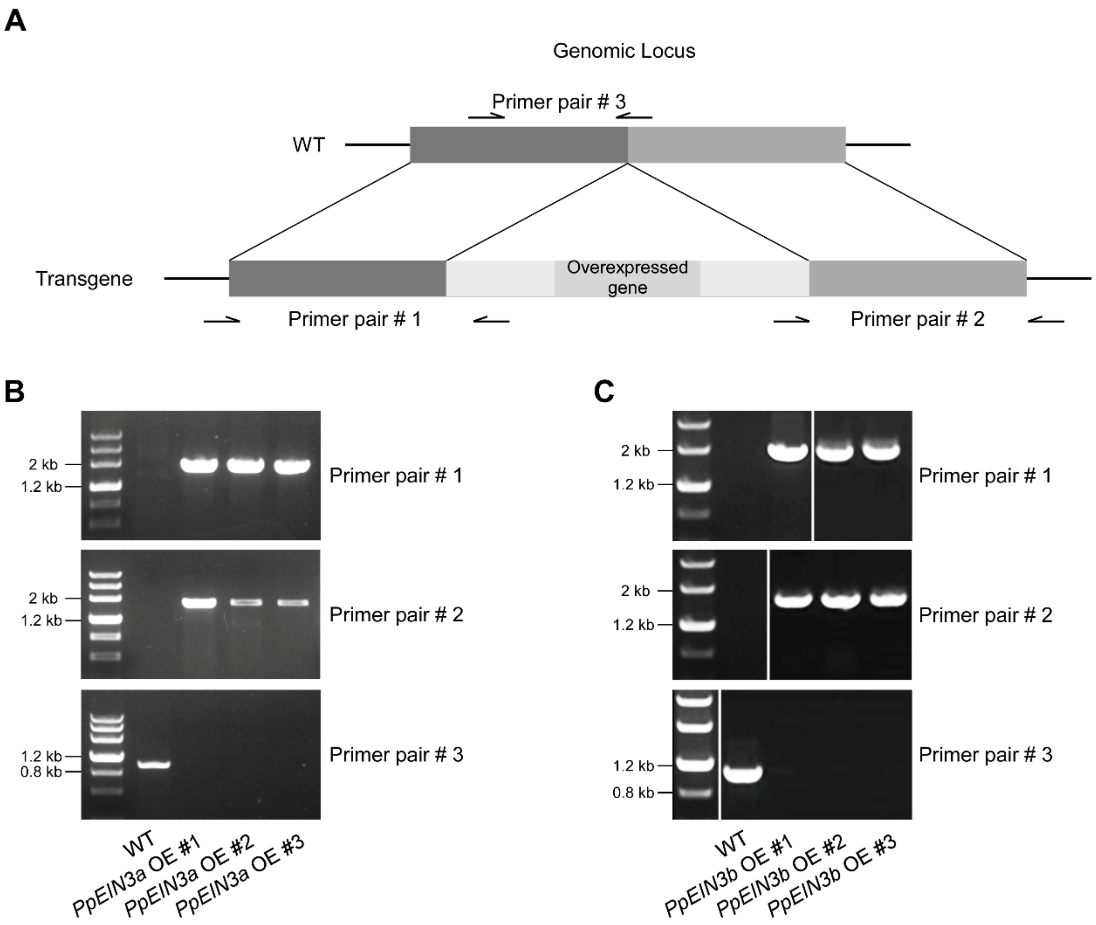

Supplementary Fig. S4. Genotyping analyses of *PpEIN3a* OE and *PpEIN3b* OE transgenic *P. patens*. (A) A diagram showing PCR-based genotyping for the integration of the putative overexpression construct at a specific genomic locus using three pairs of primers. Primer pairs #1 and #2 detect homologous recombination at the upstream and downstream arms, yielding PCR fragments of 1924 bp and 1795 bp only in transgenics. Primer pair #3 targets the replaced genomic region, producing fragments of 970 bp only in the WT. **(B)** Results for three independent *PpEIN3a* OE lines. Genomic DNA was amplified using three primer pairs (Supplementary Table S1). (**C)** Results for three independent *PpEIN3b* OE lines using three primer pairs.

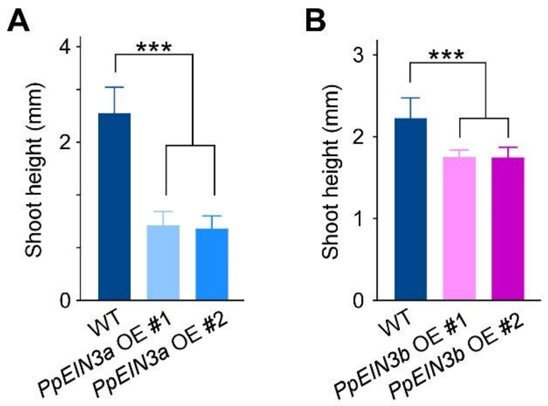

Supplementary Fig. S5. Quantification of gametophore shoot height in *PpEIN3a* OE and *PpEIN3b* OE transgenic *P. patens*. (A) Quantification of shoot height in two independent *PpEIN3a* OE lines. Data represent are mean ± SD (n = 15 gametophores). *** indicates statistical significance relative to WT at $P \leqslant 0.001$ (Student's t-test). **(B)** Results for two independent *PpEIN3a* OE lines. Data represent are mean ± SD (n = 15 gametophores). *** indicates statistical significance relative to WT at $P \leqslant 0.001$ (Student's t-test).

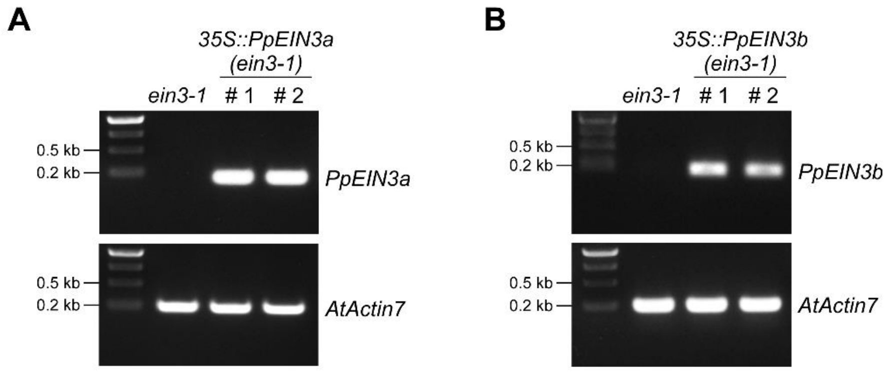

Supplementary Fig. S6. Expression analyses of *PpEIN3a* in *35S::PpEIN3a* and *PpEIN3b* in *35S::PpEIN3b Arabidopsis* transgenic lines (*ein3-1* background). (A) Detection of *PpEIN3a* transcript in *35S::PpEIN3a* lines. Seedlings were grown on half-strength MS medium for 7 days before analysis. *AtActin7* was used as an internal control. **(B)** Detection of *PpEIN3b* transcript in *35S::PpEIN3b* lines. In **(A)** and **(B)**, the expected sizes of the PCR-amplified fragments for *PpEIN3a*, *PpEIN3b*, and *AtActin7* are 138 bp, 151 bp, and 199 bp, respectively.

Supplementary table S1. Primers used in this study.

Primer name	Primer sequence (5’-3’)
**sgRNA sequence for *PpEIN3a* and *PpEIN3b* and sequencing primers for *Ppein3a/b* mutant**	
sgRNA-PpEIN3a	AACGGGTACCTCCGCTGGGG
sgRNA-PpEIN3b	GAGGTAGCGTAGGCACTCCT
PpEIN3a-Seq-F	GAGAACCCGACCCAACACCG
PpEIN3a-Seq-R	AGAACGCCCACCTTCCATGC
PpEIN3b-Seq-F	GAGTCGGAAGGAAGGTTGAG
PpEIN3b-Seq-R	CAGGAAGTGTGATAGGAGCG
***P. patens* OE transgene constructs**	
PpEIN3a OE-F	AGGCGCGCCATGGAGCGGGGTGAGGAGGTGGAATA
PpEIN3a OE-R	TCCCCCGGGCTAAGCGTAGTCTGGGACGTCGTATGGGTA TGCACCGAAGTACCATATGGGGTC
PpEIN3b OE-F	AGGCGCGCCATGGAGCGGGGAGGGGAGGTAGAATAC
PpEIN3b OE-R	CACCGGGCCCCTAAGCGTAGTCTGGGACGTCGTATGGGTA TGCACCGAAATACCATATGAAGTCCTC
**Genotyping of *P. patens* OE transgenic lines**	
Primer pair #1F	CATCCTACAGTTTGGCAGTCCT
Primer pair #1R	CGAGGTATGTAGGCGGTGC
Primer pair #2F	CTAAACCTGGAGCCCAGACG
Primer pair #2R	CTGCTGTAAGGACCGCAAGTAG
Primer pair #3F	TTACTCTTCTTCTGCAAACCGCT
Primer pair #3R	CGTCTATTCTGGAACCACACCGC
**Expression analysis of *PpEIN3* in *P. patens***	
qrt-PpEIN3a-F	ACCAGTGGCCTCGGTCTC
qrt-PpEIN3a-R	CCGCCAGGATTACCAACC
qrt-PpEIN3b-F	CGTAGGCACTCCTGGGTACC
qrt-PpEIN3b-R	CGCAAAGGGCTCGTAATCA
PpTubulin-F	GAGTTCACGGAAGCGGAGAG
PpTubulin-R	TCCTCCAGATCCTCCTCATA
***Arabidopsis* transgene constructs**	
35S-PpEIN3a-F	CGGGATCCGATGGAGCGGGGTGAGGAGGTGGAATA
35S-PpEIN3a-R	GACTAGTCTAAGCGTAGTCTGGGACGTCGTATGGGTATGCACC GAAGTACCATATGGGGTC
35S-PpEIN3b-F	CATGCCATGGGCATGGAGCGGGGAGGGGAGGTAGAATAC
35S-PpEIN3b-R	GAAGATCTCTAAGCGTAGTCTGGGACGTCGTATGGGTATGCAC CGAAATACCATATGAAGTCCTC
**Expression analysis of *PpEIN3* in *35S::PpEIN3 Arabidopsis* transgenic lines**	
At-PpEIN3a-F	GGTTCCATGGGAGTAGCGCGAT
At-PpEIN3a-R	CATGGTCTCGTCCGCCACGA
qrt-PpEIN3b-F	CGTAGGCACTCCTGGGTACC
qrt-PpEIN3b-R	CGCAAAGGGCTCGTAATCA

AtActin7-F	GGTGTCATGGTTGGTATGGGTC
AtActin7-R	CCTCTGTGAGTAGAACTGGGTGC
**Yeast two-hybrid constructs**	
PpEIN3a-AD-F	GGCCATGGAGGCCAGTGAATTCATGGAGCGGGGTGAGGAGGTGGAATA
PpEIN3a-AD-R	AGCTCGAGCTCGATGGATCCCTATGCACCGAAGTACCATATGGGGTC
PpEIN3b-AD-F	GGCCATGGAGGCCAGTGAATTCATGGAGCGGGGAGGGGAGGTAGAATAC
PpEIN3b-AD-R	AGCTCGAGCTCGATGGATCCCTATGCACCGAAATACCATATGAAGTCCTC
PpRSL1-BD-F	TATGGCCATGGAGGCCGAATTCATGGCAGGTCCAGCAGGAGCTTTATG
PpRSL1-BD-R	TATGCTAGTTATGCGGCCGCTTAGTCAGCAGAAGGCTGATTGCCGTCC
PpRSL2-BD-F	TATGGCCATGGAGGCCGAATTCATGGATGAGGCAACCAGGAGC
PpRSL2-BD-R	TATGCTAGTTATGCGGCCGCCTACTCTTTGTCGGCAGAAGGTT
**Bimolecular fluorescence complementation constructs**	
XY104-PpRSL1-F	GGTACCCGGGGATCCTCTAGAGTCGACATGGCAGGTCCAGCAGGA
XY104-PpRSL1-R	GAGCTGCACGCTGCCACCGCCGTCGACGTCAGCAGAAGGCTGATTGC
XY104-PpRSL2-F	GGTACCCGGGGATCCTCTAGAGTCGACATGGATGAGGCAACAGGAGC
XY104-PpRSL2-R	GAGCTGCACGCTGCCACCGCCGTCGACCTCTTTGTCGGCAGAAGGTTGA
XY103-PpEIN3a-F	GGTACCCGGGGATCCTCTAGAGTCGACATGGAGCGGGGTGAGGAG
XY103-PpEIN3a-R	GCCCTTGCTCACCATACCGCCGTCGACTGCACCGAAGTACCATATGGG
XY103-PpEIN3b-F	GGTACCCGGGGATCCTCTAGAGTCGACATGGAGCGGGGAGGGGA
XY103-PpEIN3b-R	GCCCTTGCTCACCATACCGCCGTCGACTGCACCGAAATACCATATGAAGTC

Supplementary materials and methods

Plant materials and growth conditions

Physcomitrium patens ecotype Gransden 2004 WT and genetic materials generated in this study were grown at 23 °C under a 16-hour light (50 μmol photon m^{-2} s^{-1})/8-hour dark cycle. Protonemal tissues were propagated on BCDAT medium, and gametophores were grown on BCD medium (Nishiyama et al., 2000), both supplemented with 1 mM $CaCl_2$, 0.5% (w/v) glucose, and 0.7% (w/v) agar.

Arabidopsis thaliana Columbia WT, *ein3-1* mutant, and transgenes lines, as well as *Nicotiana benthamiana*, were cultured at 22 °C under a 16-hour light/8-hour dark cycle.

Generation of *P. patens* disruptant mutants and OE transgenic lines and phenotypic analysis

CRISPR-Cas9 gene editing method was used to create *Ppein3a/b* mutant. The design of the sgRNA sequence for the target gene (Table S1), the subcloning of sgRNA sequence into pUC57 vector, and the information of the other plasmids used for protoplast transformation were as described (Wang et al., 2024).

To make the OE constructs, full-length CDSs (2 kb) of *PpEIN3a* and *PpEIN3b* were amplified from *P. patens* cDNA (see primers in Table S1). For *PpEIN3a* OE construct, PCR products were digested with *Asc*I and *Sma*I (introduced in primers) and ligated into the pTFH15.3 vector, yielding *PpEIN3a-pTFH15.3*, where *PpEIN3a* expression was controlled by the rice *Actin2* promoter. For *PpEIN3b* OE construct, PCR products were digested with *Asc*I and *Apa*I and cloned into pTFH15.3, producing *PpEIN3b-pTFH15.3*. Both plasmids were linearized with *Not*I before transformation.

PEG-mediated protoplast transformation and antibiotic selection followed Wang et al. (2024). *Ppein3a/b* mutant was confirmed by sequencing the genomic regions of *PpEIN3a* and *PpEIN3b* using primers (Table S1) covering the gene editing region for each gene. Positive OE transgenic lines were verified by PCR genotyping (Table S1).

As for the analysis of phenotypes in *Ppein3a/b* mutants and *PpEIN3* OE lines, the gametophore apices of uniform size were cultured on BCD medium for 6 weeks for material activation, followed by subculture for 35 days, with at least 15 gametophore apices used per genotype. A representative medium-sized gametophore was selected from each subculture for rhizoid length measurement. After the complete removal of the agar medium adhering to the materials, the rhizoids were carefully separated using sterile water, and the top 5 longest rhizoids per gametophore were measured under a stereo dissection microscope (Leica M25, equipped with LEICA APPLICATION SUIT-LAS v.4.12 software). Thus, a total of 75 rhizoids were measured for each genotype (n = 15 gametophores). The shoot height of the gametophores was also measured under the stereo dissection microscope. Experiments were repeated three times with similar trends, and representative data from one repetition are presented.

Generation of *Arabidopsis* transgenic lines and phenotypic analysis

CDSs of *PpEIN3a* and *PpEIN3b* were amplified from their respective plasmids

(Supplementary Table S1). For *35S::PpEIN3a*, PCR products were digested with *BamH*I and *Spe*I and inserted into pCAMBIA1302 using *Spe*I and *Bgl*II sites. For *35S::PpEIN3b*, products were digested with *Nco*I and *Bgl*II and cloned into pCAMBIA1302.

Arabidopsis transformation and generation of homozygous lines were performed as described in Wang et al. (2024). Seeds were surface-sterilized and germinated on half-strength MS medium for 5 days. Root hairs were imaged from ≥15 seedlings per genotype, and length was quantified using ImageJ. Experiments were repeated three times.

Gene expression analysis

Transcript levels of target genes were quantified by RT-qPCR. The extraction of RNA from moss and *Arabidopsis* and the operation of real-time PCR were described previously (Wang et al., 2024). Gene-specific primers used are listed in Table S1.

Yeast two-hybrid assay

CDSs of *PpEIN3a* and *PpEIN3b* were amplified from their plasmids (Supplementary Table S1) and cloned into pGADT7. CDSs of *PpRSL1* and *PpRSL2* were amplified from *P. patens* cDNA and cloned into pGBKT7. Yeast strain AH109 was co-transformed using PEG-mediated transformation. Protein interactions were assayed by growth on -LWH medium supplemented with 3-aminotriazole (3-AT).

Bimolecular fluorescence complementation assay

CDSs of *PpEIN3a* and *PpEIN3b* were cloned into XY103, and *PpRSL1* and *PpRSL2* into XY104 (Supplementary Table S1). Constructs were introduced into *A. tumefaciens* GV3101, mixed with P19, and co-infiltrated into *N. benthamiana* leaves. Plants were incubated in darkness for 12 hours and then grown under standard conditions for 2–3 days before undergoing laser scanning confocal fluorescence imaging (Leica STELLARIS).

Supplementary References

Nishiyama T, Hiwatashi Y, Sakakibara I, Kato M, Hasebe M. Tagged mutagenesis and gene-trap in the moss, *Physcomitrella patens* by shuttle mutagenesis. *DNA Res*. 2000:7(1):9-17.

Wang Y, Jiang L, Kong D, Meng J, Song M, Cui W, Song Y, Wang X, Liu J, Wang R, *et al.* Ethylene controls three-dimensional growth involving reduced auxin levels in the moss *Physcomitrium patens*. *New Phytol.* 2024:242 (5):1996-2010.
